# Immediate and 12 months follow up of function and lead integrity after cranial MRI in 356 patients with conventional cardiac pacemakers

**DOI:** 10.1186/1532-429X-16-39

**Published:** 2014-06-05

**Authors:** Olaf M Muehling, Reza Wakili, Martin Greif, Franz von Ziegler, Dominik Morhard, Hartmut Brueckmann, Alexander Becker

**Affiliations:** 1 Cardiology Clinic Harlaching, University of Munich, Munich, Germany; 2 Department of Medicine, University of Munich, Munich, Germany; 3 Department of Neuroradiology, University of Munich, Munich, Germany

**Keywords:** Pacemaker, Magnetic resonance imaging, Safety

## Abstract

**Background:**

Conventional cardiac pacemakers are still often regarded as a contraindication to magnetic resonance imaging (MRI). We conducted this study to support the hypothesis that it is safe to scan patients with cardiac pacemakers in a 1.5 Tesla MRI, if close supervision and monitoring as well as adequate pre- and postscan programming is provided.

**Methods:**

We followed up 356 patients (age 61.3 ± 9.1 yrs., 229 men) with single (n = 132) or dual chamber (n = 224) cardiac pacemakers and urgent indication for a cranial MRI for 12 months. The scans were performed at 1.5T. During the scan patients were monitored with a 3-lead ECG and pulse oximetry. Prior to the scan pacemakers were programmed according to our own protocol.

**Results:**

All 356 scans were completed without complications. No arrhythmias were induced, programmed parameters remained unchanged. No pacemaker dysfunction was identified. Follow-up examinations were performed immediately, 2 weeks, 2, 6, and 12 months after the scan. There was no significant change of pacing capture threshold (ventricular 0.9 ± 0.4 V@0.4 ms, atrial 0.9 ± 0.3 V@0.4 ms) immediately (ventricular 1.0 ± 0.3 V@0.4 ms, atrial 0.9 ± 0.4 V@0.4 ms) or at 12 months follow-up examinations (ventricular 0.9 ± 0.2 V@0.4 ms, atrial 0.9 ± 0.3 V@0.4 ms). There was no significant change in sensing threshold (8.0 ± 4.0 mV vs. 8.1 ± 4.2 mV ventricular lead, 2.0 ± 0.9 mV vs. 2.1 ± 1.0 mV atrial lead) or lead impedance (ventricular 584 ± 179 Ω vs. 578 ± 188 Ω, atrial 534 ± 176 Ω vs. 532 ± 169 Ω) after 12 months.

**Conclusions:**

This supports the evidence that patients with conventional pacemakers can safely undergo cranial MRI in a 1.5T system with suitable preparation, supervision and precautions. Long term follow-up did not reveal significant changes in pacing capture nor sensing threshold.

## Background

It is estimated that 50% to 75% of patients with implantable cardiac devices will need magnetic resonance imaging (MRI) at some point after implantation [1]. Despite newly developed MRI-safe devices and leads [2,3], the majority of cardiac pacemaker patients will still present with conventional cardiac pacing devices for the next decade. Thus, the presence of a permanent pacemaker is still often considered a contraindication to MRI [4], and device manufacturers warn against MRI procedures for patients with such devices [5-7]. There have been at least 17 supposed MRI-associated deaths worldwide among patients with pacemakers, but none of the deaths occurred during appropriate physician-supervised monitoring [8]. Thus redundant monitoring, relevant pre-MRI reprogramming, and specific absorption rate (SAR) management, are the cornerstones of the risk mitigation strategies when scanning device patients [9]. Recently, a position paper on this topic was published stating that on a case-by-case basis, the diagnostic benefit from MRI may outweigh the risks for some pacemaker and ICD patients [10]. Articles have been published demonstrating the relative safety of scanning patients with pacemakers [11,12]. In a larger group of patients (>400) it has been shown, that with appropriate precautions, MRI can be done safely with selected cardiac devices [13]. Because changes in device variables and programming may occur, electrophysiologic monitoring during MRI is essential. Thus, follow up studies were limited in the number of patients (n = 55) and time of follow-up after their scan (up to 99 days). We conducted this study on a large number of patients (>300) with a variety of pacemakers undergoing an urgent cranial MRI. Several follow-up exams were analyzed up to 12 months after scanning to support the hypothesis that it is safe to scan patients with cardiac pacemakers at 1.5 T, if close supervision and monitoring as well as adequate pre- and postscan programming is provided.

## Methods

Eligible subjects consisted of patients with implanted permanent pacemakers and an indication for an urgent cranial MRI indicated by their referring neurologist or neurosurgeon and no acceptable imaging alternative (due to superiority of MRI or contraindication to the alternative imaging modality). Devices must have been implanted at least 2 months prior to the MRI scan and have a battery status of "Beginning of life" (BOL). Patients who had an epicardial pacing lead or a known or suspected lead fracture were excluded. Study subjects were enrolled between July, 2004 and January 2012. The study complied with the Declaration of Helsinki and was approved by the local Institutional Review Board (Ludwig-Maximilians-University Munich, Medical Faculty).

### Study protocol

**Figure 1:**
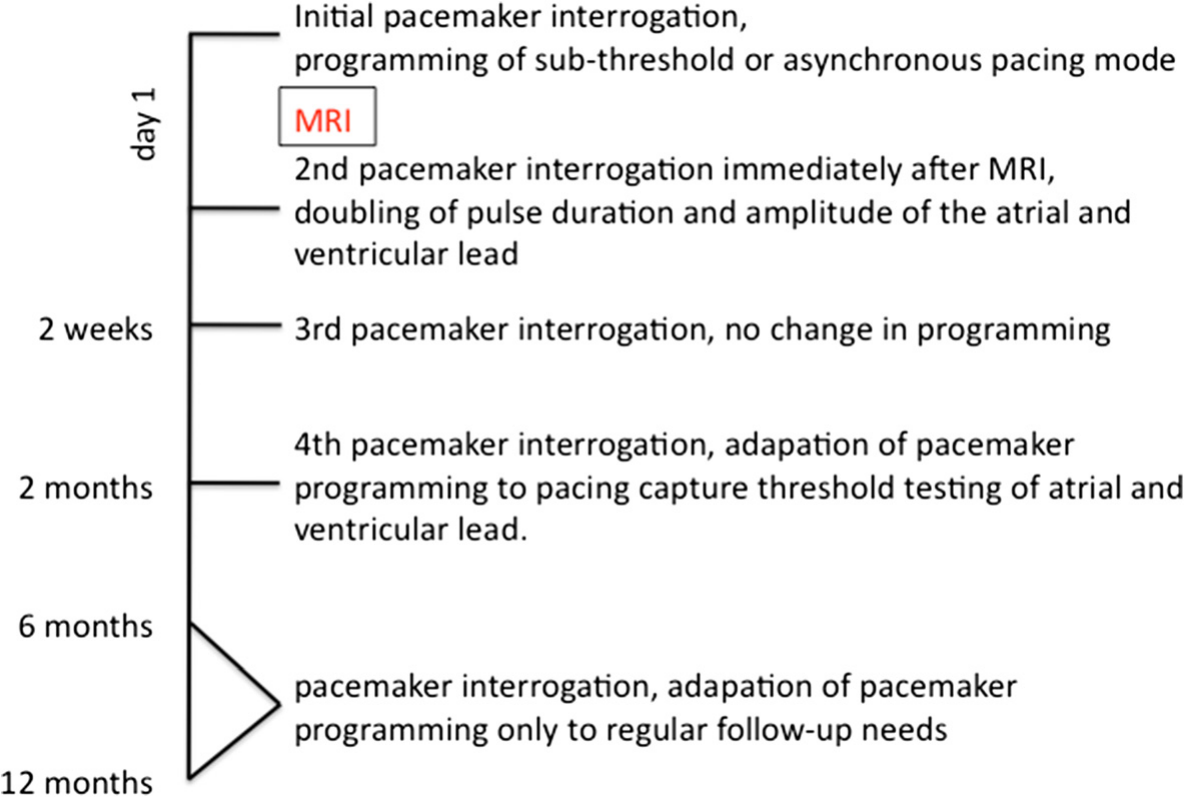
Underlying rhythm prior to MRI.

**Figure 2:**
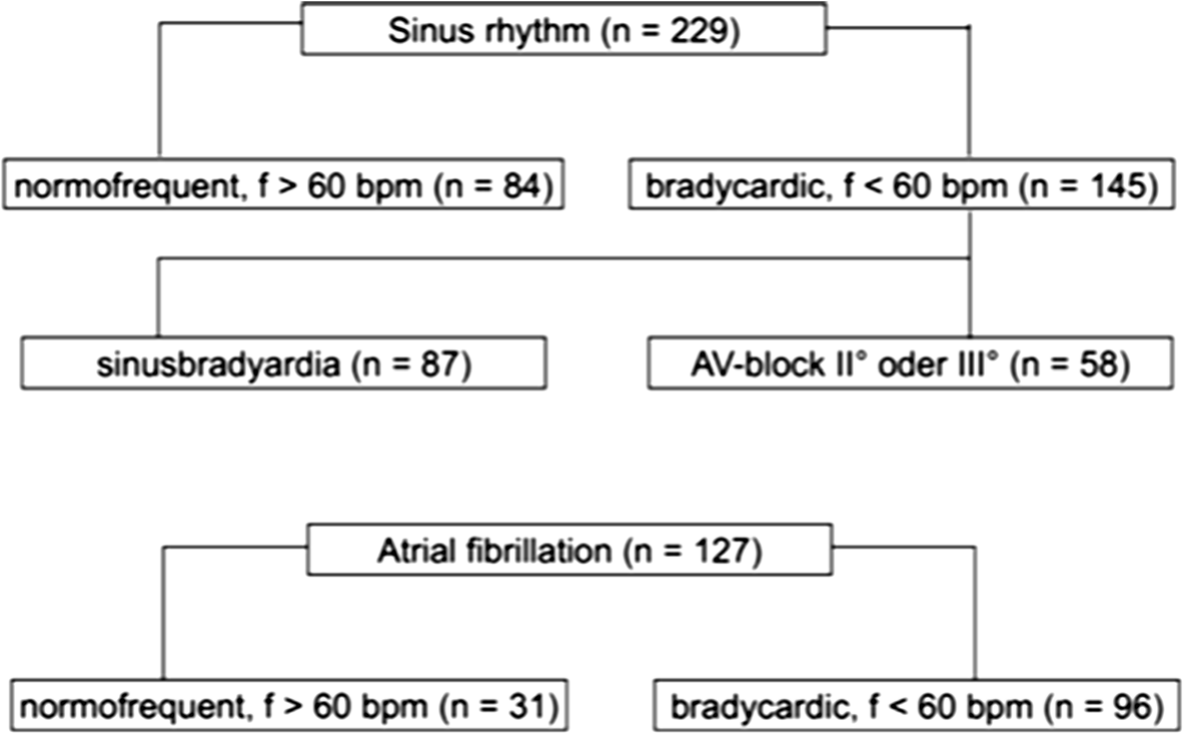
Time schedule and follow-up plan.

Prior to the scan, all participants received education regarding potential risks (irreversible device damage or malfunction, thermal injury, arrhythmias) and had to give written, informed consent to participate in the MRI scan.Underlying rhythm was confirmed (Figure 1). Full interrogation of all device information and impedance, sensing and capture function are measured immediately before and after MRI and at every follow-up exam (Figure 2). Sensing and impedance were measured using the automated features of the manufacturer’s program. After the scan, and at every follow up patients were asked for clinical symptoms. Troponin I serum levels were also obtained within 6-12 hrs. after the scan. Follow-up interrogations were performed at 2 weeks, 2, 6, and 12 months after the scan together with a screening of the pacemaker and leads for loss of capture, unexpected generator malfunction or failure, device resetting, or early battery depletion.

During the scan all pacemakers were programmed to an asynchronous stimulation mode (VOO or DOO) if the patient was pacemaker dependent, or to a subthreshold pacing without changes to the sensing parameters in non pacemaker dependent patients. If possible, magnet response, rate response, premature ventricular contraction response, noise response, ventricular sense response, conducted atrial fibrillation response, and tachyarrhythmia functions (monitoring, antitachycardia pacing) were disabled. Histograms and event logs were saved prior to scan. After the MRI scan was completed, each device was reprogrammed to its pre-scan settings. If patient tolerated, pulse amplitude and pulse duration were doubled immediately after the MRI exam according to our own security protocol to avoid ineffective stimulation due to an increase of pacing capture threshold. Pacing capture thresholds were restored to initial values at the 8-weeks-interrogation period.

All MRI scans were performed on a Siemens Symphony 1.5 T scanner. Scans were performed using usual protocols with peak SAR limited to 2 W/ kg bodyweight. Scan time was limited to 30 minutes. Turbo spin echo, FLAIR, and diffusion sequences were permitted. Continuous monitoring was performed with an in-vivo, non-invasive monitoring system including telemetry and continuous pulse oximetry with plethysmographic waveform. Blood pressure measurements were obtained every 3 minutes. A cardiologist was present for the entire scan and resuscitation equipment was available in the MRI suite. Staff was trained according to our in-house protocol reported earlier [14].

### Statistics

Statistical analysis was performed using SigmaStat for Windows (V 3.10; Systat Software GmbH; Erkrath; Germany). The Shapiro–Wilk test was used to assess normality. Analysis was performed using the paired Wilcoxon rank sum test with continuity correction for continuous variables, and the Kruskal–Wallis test for categorical data. Comparison between pre- and follow-up-scans was performed using ANOVA. A two-sided P-value of <0.05 was considered significant.

## Results

We examined 356 patients (64,3% male), mean age 61,3 ± 9,1 years with indication for an urgent cranial MRI. Indications were known or suspected cerebral tumor in 74,8%, s.p. glioblastoma resection in 16,5%, and vasculitis in 8,7%. Indications for pacemaker implant were sick sinus syndrome in 33,0%, bradicardic atrial fibrillation in 27,9%, symptomatic AV Block II° in 19,1% or III° in 20,0%. Mean time after pacemaker implantation or lead implantation at MRI examination was 40 months ± 28 months (range 2 - 97 months). Intrinsic patient rhythm assessed prior to the MRI is displayed in Figure 1. 241 patients were pacemaker-dependent. Mode and lead information are shown in Table 1. Pacemaker and lead manufactures are shown in Tables 2 and 3. Follow up was completed in 348 after 2 months, 346 after 6 months and 338 patients after 12 months.

**Table 1:** Information on pacemaker mode and lead configuration

**Mode**	**n (%)**	
AAI(R)	6 (1,7)	
VVI(R)	126 (35,4)	
DDD(R)	224 (62,9)	
**Leads**	N (%)	bipolar
Atrial	230 (64,6)	183
Ventricular	350 (98,3)	238

**Table 2:** Pacemaker models

***Pacemaker***	***n***
ELA Chorus	6
Guidant Insignia (Entra, Plus)	12
Medtronic Kappa	103
Medtronic Enpulse	83
Medtronic Thera	12
Medtronic Sigma	6
Pacesetter Affinity	9
Pulsar Max	3
St.Jude Verity	15
St. Jude integrity	6
Vitatron (Selection, 9000, Adapta, T70DR)	101

**Table 3:** Leads

***Leads***	***n***
Medtronic	344
Biotronic	12
Osypka	9
Pacesetter	25
St.Jude	6
Vitatron	21
Sorin	155
ELA	8

All 356 scans were completed without immediate complications. No clinical symptoms consistent with device movement, torque, heating palpitations or dizziness occurred during MRI examinations. No hemodynamic relevance of asynchronous stimulation was monitored. No procedures were terminated due to clinical events or patient complaints. Diagnostic questions were answered in all sequences, despite the unavoidable artifacts due the pacemaker or leads.

All devices were functioning appropriately after MRI. Although in 37 devices (10,4%) Power- on-Reset occured and in some reprogramming was necessary: Medtronic (Thera n = 6, Sigma n = 4), Pacesetter (n = 6), Pulsar (n = 2) partial or full reset occurred but battery voltage remained stable and battery impedance remained unchanged or slightly increased (no reprogramming necessary); St.Jude integrity/verity (n = 9), Vitatron T-series (n = 10) ERI was triggered and the ERI message could be cleared with the programmer to normal function after the scan.

**Figure 3:**
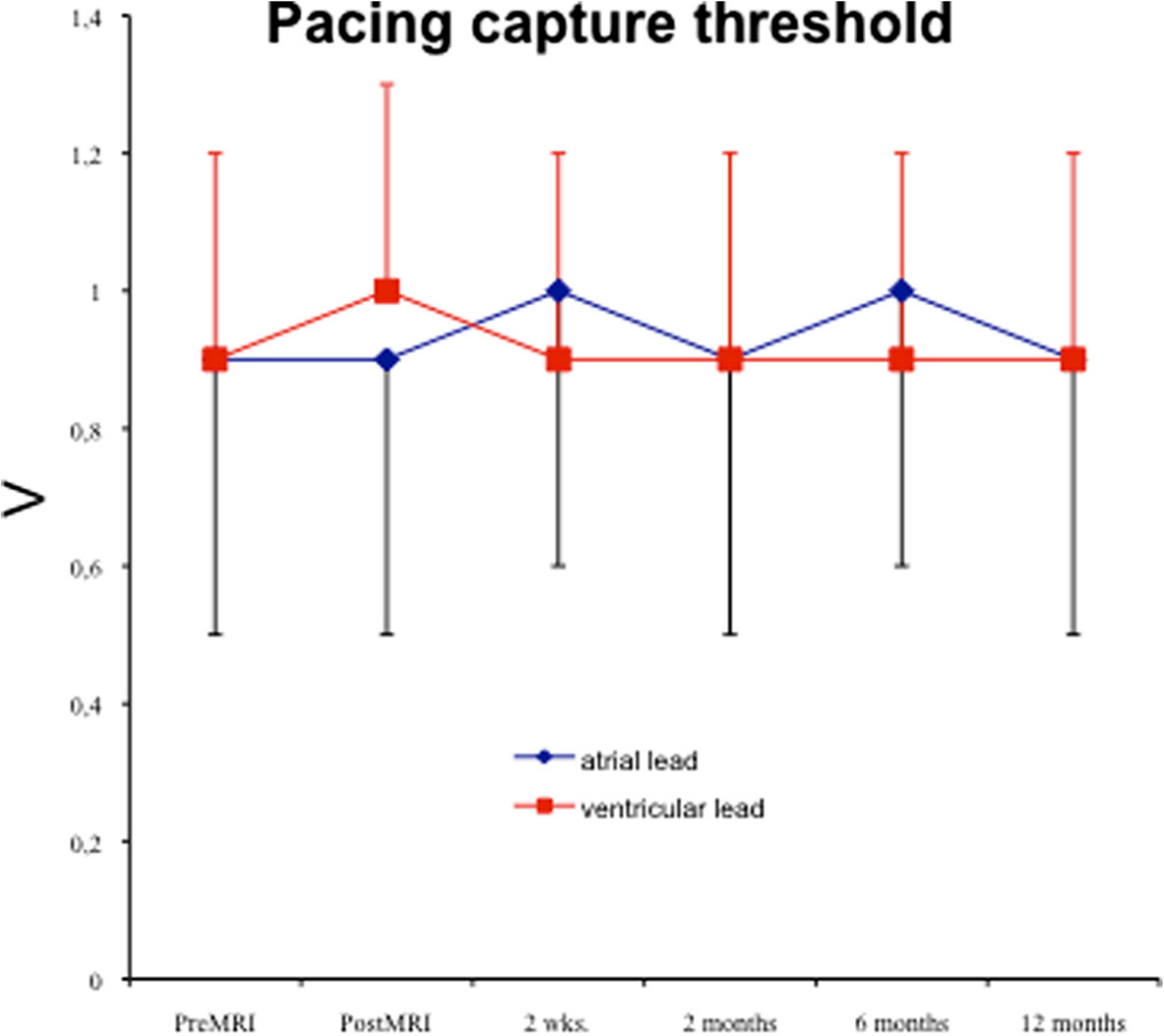
Pacing capture threshold for atrial and ventricular leads prior and post MRI and during 2 weeks (n = 356), 2 (n = 354), 6 (n = 346), and 12 months follow-up (n = 338).

**Figure 4:**
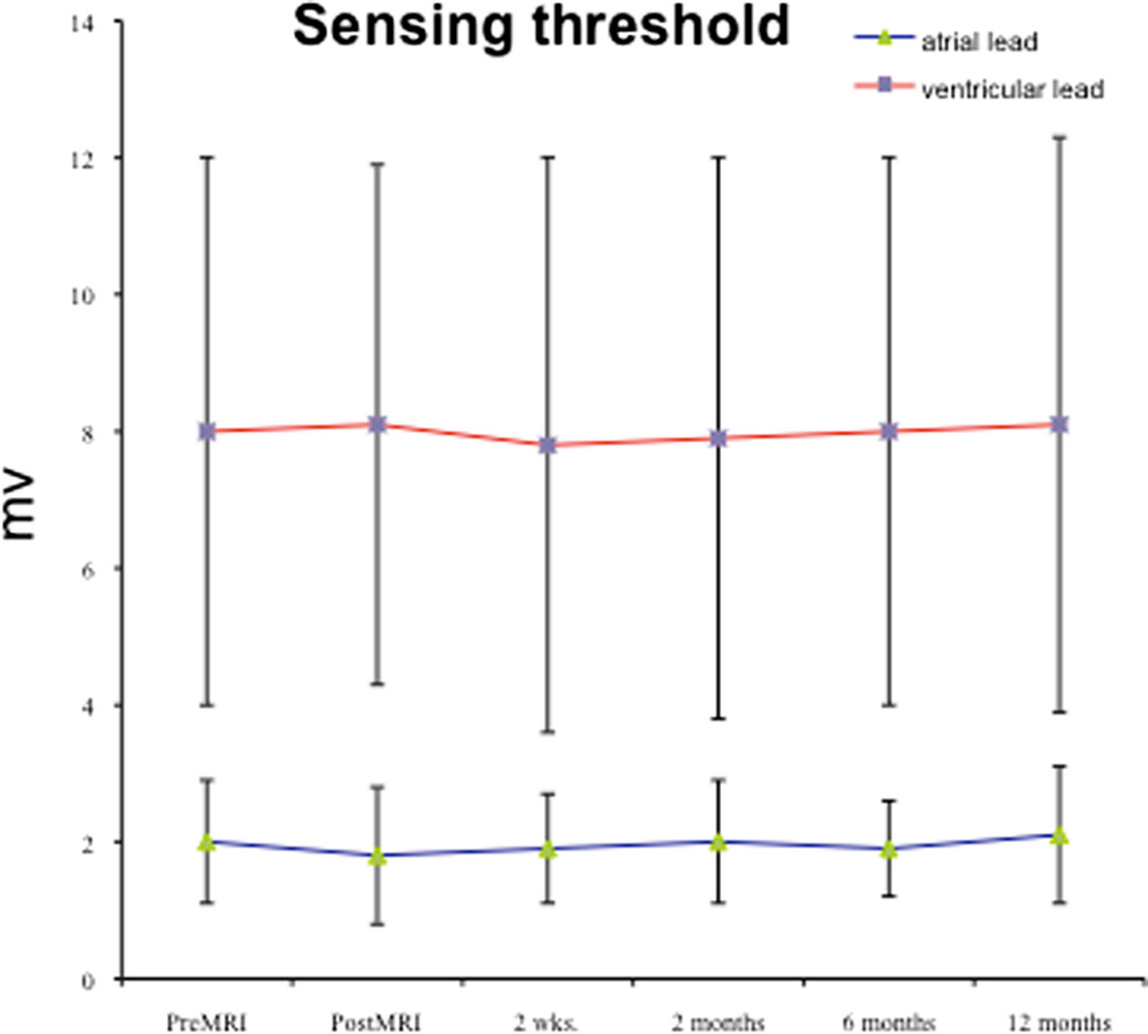
Sensing threshold for atrial and ventricular leads prior and post MRI and during 2 weeks (n = 356), 2 (n = 354), 6 (n = 346), and 12 months follow-up (n = 338).

**Figure 5:**
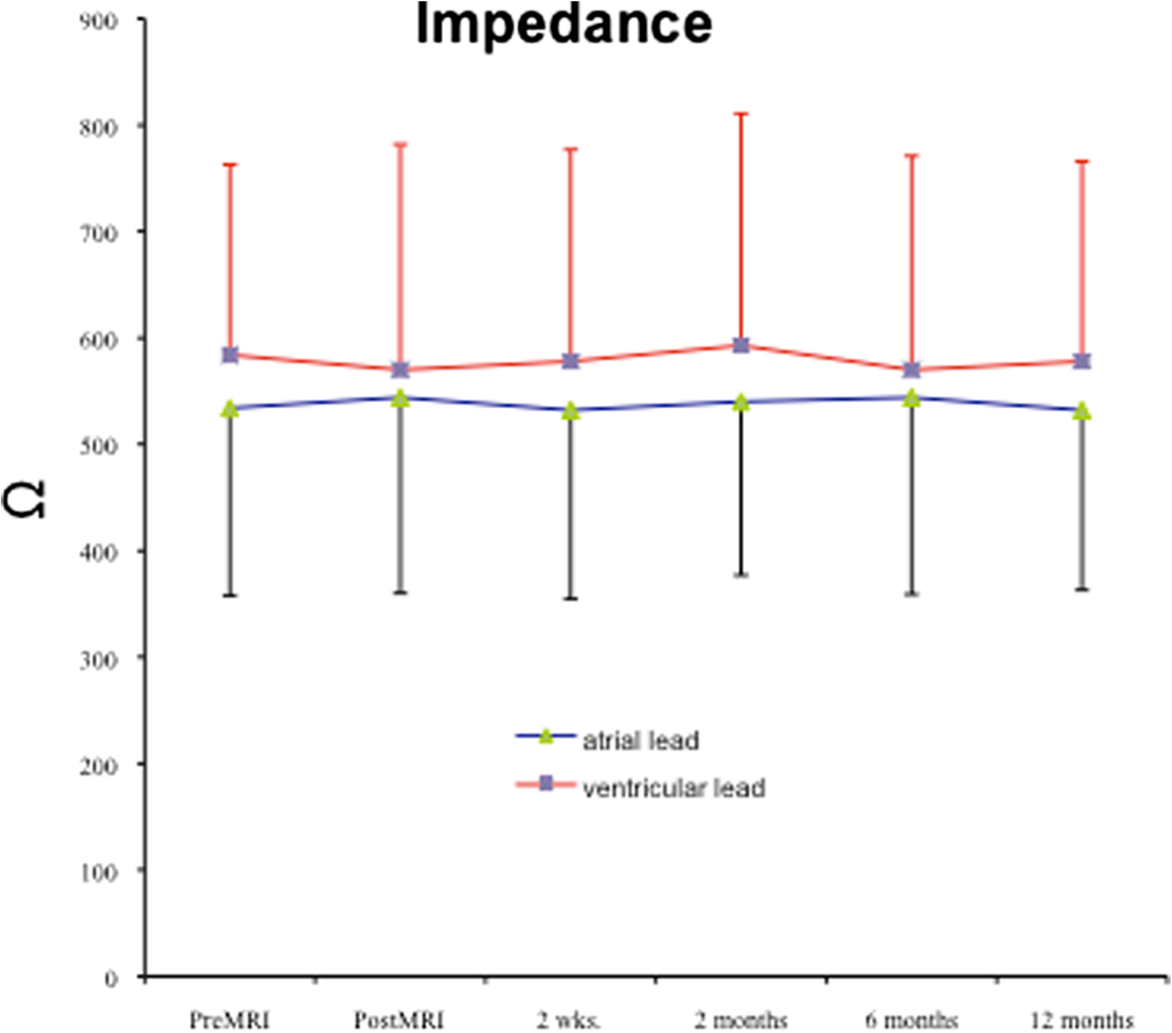
Impedance for atrial and ventricular leads prior and post MRI and during 2 weeks (n = 356), 2 (n = 354), 6 (n = 346), and 12 months follow-up (n = 338).

In 310 of 356 patients no change in pacing threshold was observed. In 27 patients a maximum increase of 0.2 V and in 19 patients a maximum increase of 0.4 V was observed. The maximum relative increase in these patients was 40%. Thus also in these patients the increase was below the recommended safety margin of a double threshold (or 200%) for pacing capture or sensing. consequently, there was no need to permanently increase output or sensing threshold after MR procedure other than we recommended in our protocol for safety reasons.No immediate or late pacemaker dysfunction was registered. There was no increase in Troponin I level within 12 hours after the MRI. Programmed parameters remained unchanged. Data for pacing capture, sensing threshold and lead impedance at 2 weeks, 2, 6, and 12 months after the scan are displayed in Figures 3, 4 and 5. At no time a significant increase in pacing capture threshold or sensing threshold could be observed. An increase in lead impedance was not registered. Increased pulse duration and pulse amplitude could be restored to initial programming in all patients at the 8-weeks-interrogation.

## Discussion

Although it has been shown that MRI can be safely performed in patients with selected conventional implantable cardiac devices without serious adverse events [12], MRI is currently unavailable for a large proportion of patients with permanent pacemaker. A recent risk assessment of pacemaker patients who undergo MRI in a 1.5 T scanner in comparison to a control group of pacemaker patients who did not undergo MRI showed no deaths, device failures requiring generator or lead replacement nor arrhythmias. Observed were minimal decreases in battery voltage. Although, statistically significant differences between the MRI and control groups for the change in pacing lead impedance and pacing threshold were seen, these differences were not clinically relevant [15].

The results of the present study show that cranial MRI can be performed safely using a protocol that incorporates device programming to minimize inappropriate activation or inhibition of bradyarrhythmia therapies, and limitation of the estimated whole-body averaged SAR of MRI sequences without additional device selection. This is in agreement with a recent smaller study [16]. As an additional security measure, we temporarily increased pacing capture parameters for a period of 2 months after the scan. To the best of our knowledge, our current study shows the largest source of data and the longest follow up (12 months) in an unselected patient (n = 356) and device population undergoing cranial MRI in a 1.5 Tesla scanner. Patients remained asymptomatic during the scan. Furthermore, no clinical relevant changes in pacing and sensing parameters was recorded. Our protocol excludes device leads that are prone to heating owing to lack of cooling by blood flow. Neither clinical heating nor significant change in pacing threshold (>1 V) as an indirect sign of thermal injury of the electrode–tissue boundary was identified. Newer devices contain only a limited amount of ferromagnetic material. As a result, the force and torque are limited, varying between 0.05 and 3.6 Newton for pacemakers. For reference, forces <2 N will not be felt by patients [17]. Lead tips are never moved magnetically as they do not possess ferromagnetic materials [18,19].

We and others plead for limiting SAR as an additional safety measure [20]. The current scientific statement from European Society of Cardiology recommends a SAR limit of 2 W/kg [10]. However, earlier data using SAR of up to 2 W/kg showed significant pacing threshold changes occurred in 9.4% of patients [11]. In a series with a 1.5 T scanner with peak SAR limited to 1.5 W/kg significant increases in pacing thresholds were seen in only 3.1% of leads [21] and no significant adverse events were seen in another study when peak SAR was kept to <2.0 W/kg [12]. We purposely used a SAR limited to 2 W/kg bodyweight for safety reasons, and we did not observe a significant change of pacing threshold. Irrespective of peak SAR level, preparations must be in place to address potential device failure during or following a scan.

Pacing threshold changes have been attributed to heating at the lead-tissue interface. In vitro models have detected local tissue heating up to 23.5°C during exposure of a pacing system to the MRI environment [22]. Temperature changes were found to a maximum of 45.9°C at the lead tip with a 1.5-T scan [23]. Animal studies evaluated local tissue heating with a peak SAR set to 3.8 W/kg [24] and found no evidence of tissue necrosis at post-scan necropsy. Although MRI of the head and lumbar regions at 1.5 T had temperature changes ≤ 0.5°C [25], it has been recognized that the lead lengths (the longer, the higher) and position (more in the right than in the left hemithorax) will affect temperature changes with the highest temperatures being at the tip of the ventricular electrode [26,27]. Furthermore, it has been demonstrated that abandoned leads exhibit greater lead tip heating compared to pacemaker attached leads [28].

In the present study, no significant immediate or long-term changes in device or lead parameters were observed. The lack of any significant changes in device parameters in the present study may be related to avoidance of nontransvenous epicardial leads, limitation of estimated SAR, or improved electromagnetic interference protection in more modern devices.

There have been reported changes in reed switch activity during an MRI examination [12,29]. Reed switch operation can result in asynchronous pacing [30], with an increased likelihood of induction of arrhythmias. Reed switch action are unpredictable, depending on the strength of the magnetic fields [19]. In our study approximately 15% of patients had reed switch activation with asynchronus stimulation in patients with spontaneous heart rate of > 60/min. No clinically relevant events occurred during scanning.

The use of asynchronous pacing in non-pacemaker-dependent patients during the scan has been criticized [31,32] due to induction of ventricular arrhythmias. 0V0, 0D0, 000, or subthreshold programming were proposed as an alternative. We and others [33] believe that induction of tachyarrhythmias during asynchronous pacing is extremely rare and our strategy followed current general recommendations [10]. Concerns about the potential for induction of ventricular fibrillation during asynchronous pacing in patients with a spontaneous rhythm, have made the use of continuous ECG telemetry and the availability of resuscitation personnel and equipment imperative [34]. There is controversy about reprogramming the pacemaker before the MRI examination [35,36]. Although the American Heart Association and American College of Radiology do not consider it necessary to reprogram the pacemaker, many studies regarding MRI protocol among patients with pacemakers require pacemakers to be placed in an asynchronous mode before imaging.

### Limitations

The current study cannot be extrapolated to 3 T MRI scanners. Furthermore, it cannot be used to risk stratify recently implanted systems or to generators at ERI or EOL. As no CRT or ICD devices were included, conclusions regarding this subgroup of devices cannot be drawn from our study.

According to our and others findings, the data on MRI are heterogeneous, and a definitive statement cannot be made about imaging patients with pacemakers. In particular in patients with older devices, unpredictable changes in device behavior can occur [37], which stresses the need for close monitoring during, and careful device interrogation after scanning.

The MRI environment is dynamic. Multiple interrelated factors may influence the risk in the MRI scanner. This includes not only SAR level and body landmark, but also MRI manufacturer, generator and lead system manufacturer and model [17]. The proximity of the generator to the end of the bore, proximity of the generator to the surface of the bore, body habitus, lead geometry and loops as well as lead system integrity [38,39] may also play a role. There may be an unpredictable change in pacemaker function such as inhibition, rapid ventricular pacing occur [30] or no change at all [40]. Furthermore there can be switching to demand mode, ventricular backup pacing activation, and electrical reset [41]. Although lead and implant geometry may play a role in lead heating [26], these variables were not included in the observation of the study.

## Conclusion

MRI of patients with pacemakers may be considered with caution, and with the benefits outweighing the risks of the examination. The presence of a permanent pacemaker no longer represents a strict contra-indication to MR in carefully selected clinical circumstances provided that specific strategies are followed. Regarding lead heating concerns and possible scar formation around the lead tip, we consider that a last pacemaker check-up 2 months after the scan should be sufficient. Until then, any scar formation would be completed and any increased in pacing threshold should have occurred. We speculate if the patient is appropriately monitored and SAR limits are respected, that scan times over 30 minutes will not result in significant other changes to the device parameters than we observed.

To date, no known deaths have been reported of patients with pacemakers or implantable cardioverter-defibrillators (ICD) undergoing MRI under the supervision of a physician while on telemetry.

Although, given the infinite possibilities of electromagnetic interferences and data published previously, the absolute safety of pacemakers and ICD and MRI interactions cannot be assured. While the results are encouraging, one should not assume that MRI may be performed without limitations.

## Abbreviations

MRI: Magnetic resonance imaging; ECG: Electrocardiogram; SAR: Specific absorption rate; FLAIR: Fast low angle inversion recovery; ICD: Implantable cardioverter defibrillator; EOL: End of life; CRT: Cardiac resynchronization therapy.

## Competing interests

The authors declare that they have no competing interests.

## Authors’ contributions

OM conception and design, acquisition of, analysis and interpretation of data; drafting of the manuscript. RW acquisition of data, revising the manuscript critically. MG acquisition of data, revising the manuscript critically. FZ acquisition of data, revising the manuscript critically. DM conception and design, drafting of the manuscript. HB conception and design, drafting of the manuscript. AB conception and design, acquisition of, analysis and interpretation of data; All authors participated sufficiently in the work to take public responsibility for appropriate portions of the content and have given final approval of the version to be published and agree to be accountable for all aspects of the work in ensuring that questions related to the accuracy and integrity of the work are appropriately investigated and resolved.

## Authors’ information

Muehling: Follow up of cardiac pacemakers after cranial MRI.
